# Intracranial extramedullary relapse of acute myeloid leukemia presenting as myeloid sarcoma mimicking meningioma: a case report and literature review

**DOI:** 10.3389/fonc.2026.1769002

**Published:** 2026-07-10

**Authors:** Yuan Cao, Peng Cao

**Affiliations:** Department of Neurosurgery, General Hospital of Northern Theatre Command, Shenyang, China

**Keywords:** acute myeloid leukemia, case report, differential diagnosis, extramedullary relapse, FLT3-ITD mutation, intracranial, meningioma, myeloid sarcoma

## Abstract

**Background:**

Myeloid sarcoma (MS) is a rare extramedullary tumor of immature myeloid cells. Intracranial MS occurring as an extramedullary relapse without concurrent systemic acute myeloid leukemia (AML), is exceptionally rare and poses a considerable diagnostic challenge due to its non-specific clinical and radiological features, often mimicking more common intracranial neoplasms like meningioma.

**Case presentation:**

We report a case of isolated intracranial MS in a 61-year-old female with a prior history of FLT3-ITD mutated AML (M2), who had been in sustained complete hematologic remission for four years. She presented with a two-week history of diminished responsiveness and apathy. Cranial MRI revealed a well-defined, homogenously enhancing left frontal mass with a dural tail sign and significant peritumoral edema, initially suggestive of a meningioma. However, diffusion-weighted imaging (DWI) demonstrated restricted diffusion. The tumor was resected. Histopathological examination revealed diffuse sheets of immature myeloid cells. Immunohistochemistry was positive for CD117, CD34, and CD68, with weak MPO expression, confirming the diagnosis of MS. Post-operatively, the patient was referred for systemic chemotherapy.

**Conclusion:**

This case illustrates that intracranial MS can be a form of extramedullary relapse in AML patients even during long-term remission and can closely mimic a meningioma radiologically. Key diagnostic clues include a prior history of AML and radiographic features such as restricted diffusion on DWI and prominent peritumoral edema. However, these findings are non-specific, and a definitive diagnosis relies on histopathological and immunohistochemical analysis. The management of MS should be based on systemic AML therapy principles rather than surgery alone. This report highlights the necessity of considering MS in the differential diagnosis of new intracranial masses in patients with a history of AML to ensure timely and appropriate treatment.

## Introduction

Myeloid sarcoma (MS), also known as granulocytic sarcoma, is an extramedullary solid tumor composed of immature myeloid blasts. The term “chloroma” originates from the tumor’s characteristically greenish appearance on its freshly cut surface, attributed to the presence of myeloperoxidase. MS commonly involves bones, periorbital tissues, lymph nodes, and skin. Central nervous system (CNS) involvement is rare, occurring in approximately 0.4% of leukemia patients, with isolated intracranial MS being even rarer (1). The diagnosis of MS is challenging, particularly when it occurs in isolation without a known history of hematologic malignancies or during the remission phase of such malignancies. Consequently, isolated MS is frequently misdiagnosed.

We present a rare case of intracranial MS occurring as an extramedullary relapse involving the CNS, manifesting as a left frontal lobe mass in a patient in remission from acute myeloid leukemia (AML). We detail the diagnostic and therapeutic course of this patient and provide a review of the relevant literature to enhance the understanding of this disease entity.

## Case report

In June 2025, a 61-year-old female patient was admitted to our hospital with a two-week history of progressive cognitive decline, characterized by diminished responsiveness, apathy, incoherent speech, and dysfluency.

Cranial magnetic resonance imaging (MRI) revealed an oval-shaped mass in the left frontal region, measuring approximately 5.4×4.9×5.1 cm. The lesion appeared isointense on T1-weighted images (T1WI) and slightly hyperintense on T2-weighted images (T2WI), accompanied by significant peritumoral edema. Contrast-enhanced scans showed marked tumor enhancement with a “dural tail sign”. Notably, diffusion-weighted imaging (DWI) demonstrated restricted diffusion (**Figure 1**).

**Figure 1:**
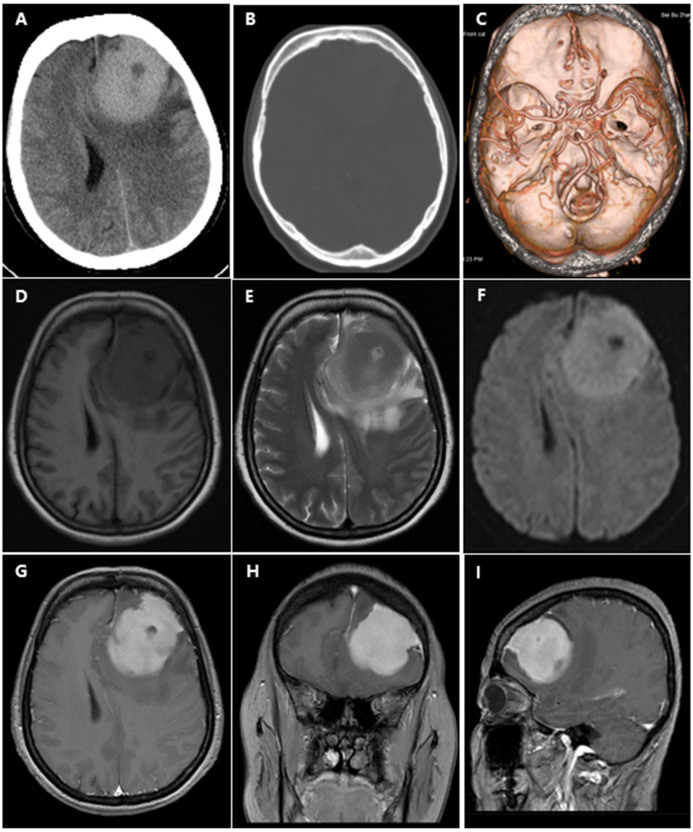
Neuroimaging findings. **(A)** Non-contrast CT (brain window). **(B)** Non-contrast CT, bone window, showing no hyperostosis or destruction. **(C)** CT angiography (CTA) demonstrating displacement of the anterior cerebral artery to the contralateral side, with no evident tumor supply from the internal carotid artery system. **(D, E)** Axial T1-weighted and T2-weighted MRI showing a well-defined mass that is isointense on T1 and slightly hyperintense on T2, with significant peritumoral edema. **(F)** Diffusion-weighted image (DWI) showing hyperintensity, indicating restricted diffusion. **(G–I)** Contrast-enhanced T1-weighted MRI showing strong tumor enhancement and a “dural tail sign”.

The patient had been diagnosed in 2021 with acute myeloid leukemia (AML-M2) harboring an FLT3-ITD mutation (**Figure 2A**; **Supplementary Figures 1**–**3**). She received sequential chemotherapy regimens including DA (daunorubicin + cytarabine), DNR + IDAC (daunorubicin + intermediate-dose cytarabine), and HA (homoharringtonine + cytarabine). She also received intrathecal chemotherapy (methotrexate 15 mg + cytarabine 75 mg + dexamethasone 5 mg) as central nervous system (CNS) prophylaxis. Bone marrow examination confirmed complete remission, a state which had been sustained for four years prior to the current admission.

**Figure 2:**
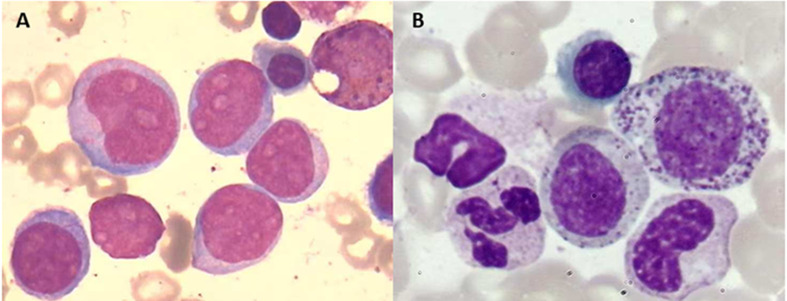
Bone marrow cytology findings of the patient. **(A)** Bone marrow cytology at initial diagnosis; **(B)** Bone marrow cytology on current admission.

On current admission, her complete blood count (CBC) was within normal limits (WBC: 5.0×10^9^/L, RBC: 4.23×10¹²/L, Hb: 135 g/L, PLT: 121×10^9^/L), and bone marrow aspiration showed no significant abnormalities, consistent with sustained complete remission (**Figure 2B**).

Given the patient’s history of AML and the radiological features suggestive of a meningioma, combined with the significant mass effect, surgical resection was pursued for both diagnostic confirmation and therapeutic decompression. Craniotomy revealed a well-defined, fungiform, solid tumor invading the dura mater. The freshly cut surface appeared grayish-white. Gross total resection was achieved, and no invasion of the underlying cerebral gyri and sulci was observed (**Figure 3**). Histopathological examination revealed a proliferation of small, round tumor cells (**Figure 4**). Immunohistochemically, the tumor cells were CD117 (+), CD34 (+), CD68 (+), and MPO (±), a profile consistent with myeloid lineage differentiation. CD56 was only focally positive, and the Ki-67 proliferation index was approximately 80%. The patient recovered well postoperatively, and she was subsequently transferred to the hematology service for systemic chemotherapy.

**Figure 3:**
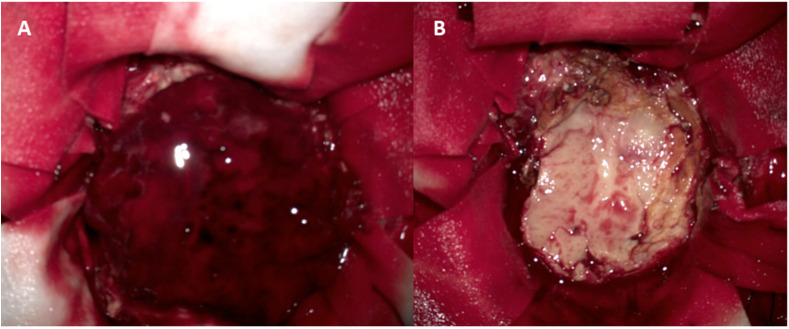
Intraoperative tumor appearance. **(A)** The tumor appears as a fungiform, solid mass. **(B)** The cut surface of the tumor is grayish-white.

**Figure 4:**
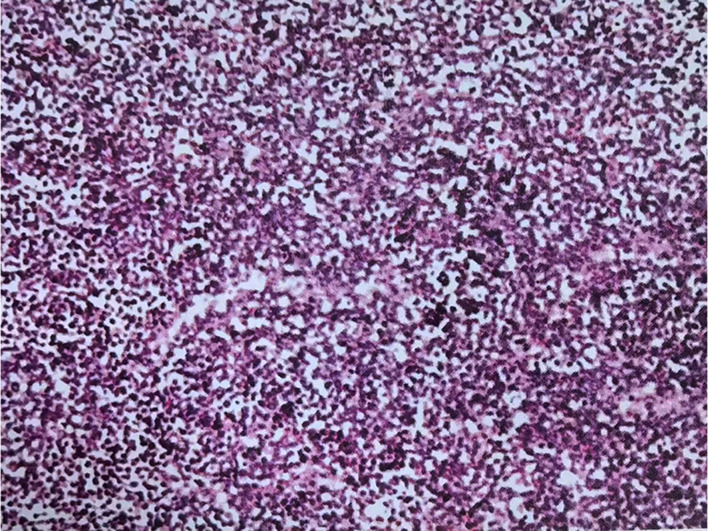
Histopathological features. The tumor is composed of diffuse sheets of relatively uniform, small round cells with large, round nuclei, prominent nucleoli, and readily identifiable mitotic figures (original magnification ×10).

## Discussion

We present a rare case of intracranial myeloid sarcoma (MS) manifesting as a CNS-restricted extramedullary relapse in a patient with FLT3-ITD-mutated AML after an exceptionally long period (four years) of complete hematologic remission. The clinical and radiographic presentation closely mimicked a meningioma, thereby presenting a considerable diagnostic and therapeutic challenge. This case is particularly instructive for three reasons: first, it demonstrates that extramedullary relapse can occur after a remission period far beyond the typical timeframe; second, it highlights the diagnostic pitfall posed by MS when it radiologically mimics a meningioma; and third, it underscores the limitations of standard CNS prophylaxis in completely eradicating sanctuary-site disease. Below, we discuss the key aspects of this case in the context of the existing literature.

### Epidemiological characteristics

The clinical course of our patient—developing isolated intracranial MS after four years of sustained remission—is exceptional when viewed against the reported epidemiological data. The reported incidence of MS varies across studies, primarily due to differences in study populations, diagnostic criteria, and follow-up durations. Among adults with AML, MS has a reported incidence of approximately 2.5-9.1% (3, 4). In pediatric AML, its frequency is higher, reaching 15-20% (5). MS may occur prior to, concurrent with, or as a form of relapse following a diagnosis of AML (6). However, the occurrence of an isolated intracranial relapse after such a prolonged remission—far beyond the median time to relapse for AML, which typically occurs within two years—is rarely documented. This exceptional timing suggests that MS can represent a very late event in the disease trajectory, potentially driven by a distinct subclone of leukemic cells that has persisted in a sanctuary site like the CNS. Intracranial MS can involve the dura mater, leptomeninges, brain parenchyma, or ventricular system (7); with subdural involvement, as seen in our patient, being particularly uncommon. This anatomical predilection in our patient directly contributed to the significant mass effect and the clinical presentation of cognitive decline, highlighting the critical importance of neuroimaging surveillance even in long-term remission.

### Pathogenesis and molecular mechanisms

The development of myeloid sarcoma (MS) is a complex, multi-step process involving dysregulated interactions between leukemic cells and the bone marrow microenvironment. Physiologically, hematopoietic stem and progenitor cells are retained in the bone marrow niche through specific adhesion molecules interacting with stromal cells (8). Key mechanisms implicated in MS pathogenesis include:

- Aberrant Adhesion Molecule Expression: Abnormal expression of adhesion molecules (e.g., CD56, CD15s, VLA-4 [α4β1 integrin]) on leukemic cells weakens their adhesion to bone marrow stromal cells, promoting extramedullary migration (9, 10). CD56 expression, in particular, is strongly associated with CNS MS. Notably, in our patient, weak CD56 positivity was observed in the current surgical specimen, whereas flow cytometry performed four years prior had been negative. Notably, in our patient, weak CD56 positivity was observed in the current surgical specimen by immunohistochemistry, whereas flow cytometry performed on bone marrow at initial diagnosis four years prior had been negative. This acquisition of CD56 expression over time suggests a possible clonal evolution favoring extramedullary migration and CNS homing, a phenomenon that has been previously described but is rarely documented with such a clear temporal distinction.
- Chemokine Receptor Dysregulation: The CXCR4/SDF-1 axis is critical for hematopoietic cell homing. Overexpression of the chemokine receptor CXCR4 or constitutive activation of its downstream signaling in leukemic cells may promote migration to extramedullary sites (11).
- Extracellular Matrix Remodeling: Upregulation of matrix metalloproteinases (MMPs), including MMP-2 and MMP-9, enhances the invasiveness of leukemic cells, enabling them to cross vascular and tissue barriers (12).
- Specific Genetic Alterations: Certain cytogenetic and molecular abnormalities are associated with an increased risk of MS. These include core-binding factor AML karyotypes such as t(8;21) and inv(16), as well as t(9;11) (13). NPM1 mutations are also linked to MS, particularly with skin and soft tissue involvement (14).

Our patient harbored an FLT3-ITD mutation, one of the most frequent genetic alterations in AML, occurring in approximately 30% of cases (15). The FLT3-ITD mutation confers a potent proliferative and survival advantage to leukemic cells via constitutive activation of downstream pathways, including PI3K/AKT, MAPK, and STAT5. Although a direct causal link between FLT3-ITD and MS remains to be fully established, the proliferative and anti-apoptotic signals driven by FLT3-ITD are likely contributors to the extramedullary disease phenotype (16).Our case adds to the growing body of evidence that FLT3-ITD-mutated AML may have a particular propensity for extramedullary involvement, especially in the CNS, and suggests that prolonged FLT3-driven clonal persistence may underlie very late relapses.

### Clinical manifestations

The clinical presentation of MS depends on the site of involvement and lacks specific symptoms. The most frequently involved sites include the skin and soft tissues (approximately 28%), lymph nodes (approximately 26%), gastrointestinal tract (approximately 16%), and bones (approximately 12%) (17). The clinical features of intracranial MS are primarily determined by the mass effect and the specific location of the tumor. Symptoms such as headache, nausea, vomiting, and papilledema constitute the most common symptom complex. Focal neurological deficits varying with the tumor location manifest as limb weakness, paresthesia, aphasia, or visual field defects. Seizures, including focal or generalized types, occur in approximately 20-30% of patients with intracranial MS (18). Our patient’s symptoms, including reduced responsiveness and apathy, are commonly associated with frontal lobe or deep-seated structure involvement. When the tumor involves the skull base or brainstem, corresponding cranial nerve palsies may occur.

Notably, approximately 25-40% of MS patients have normal peripheral blood and bone marrow findings at diagnosis, a condition termed “isolated” or “aleukemic” MS (19). Without appropriate treatment, nearly 100% of these patients will eventually progress to systemic AML within 6 to 24 months (20).Our patient, however, differs from this classical “isolated” MS population, as she had a well-documented prior history of AML. Her presentation with normal peripheral blood and bone marrow despite the presence of an intracranial mass represents a formed fruste of extramedullary relapse, reinforcing the concept that MS can be the first and only manifestation of disease recurrence, even in patients with a known history of AML.

### Imaging features

The imaging features of MS are diverse and nonspecific, frequently leading to misdiagnosis. The characteristic findings vary by modality. On non-contrast computed tomography (CT), MS typically presents as an iso- to hyperdense mass with well- or ill-defined margins, often accompanied by peritumoral edema and mass effect. On magnetic resonance imaging (MRI), lesions are typically isointense or slightly hypointense on T1-weighted images (T1WI) and heterogeneously isointense or hyperintense on T2-weighted images (T2WI) (21). Fluid-attenuated inversion recovery (FLAIR) sequences commonly show prominent peritumoral hyperintensity (edema). Most lesions demonstrate marked homogeneous enhancement after contrast administration. On diffusion-weighted imaging (DWI), lesions typically show hyperintensity with a correspondingly low apparent diffusion coefficient (ADC), indicating restricted diffusion, which is a key feature aiding differential diagnosis (22, 23). Positron emission tomography/computed tomography (PET/CT) is valuable for staging and detection of occult disease, as MS lesions typically exhibit intense ^18^F-fluorodeoxyglucose (^18^F-FDG) avidity (24).

In the present case, the principal diagnostic challenge was the lesion’s close resemblance to a meningioma. However, two details provided critical diagnostic clues. First, as a highly cellular malignant tumor, MS often exhibits restricted diffusion on DWI and significant peritumoral edema, features that are not prominent in classic meningiomas. Second, and more importantly, the patient’s history of AML was a pivotal clue. The combination of a history of AML, restricted diffusion on DWI, and significant peritumoral edema strongly suggested the diagnosis of MS over a benign meningioma. This case highlights the value of incorporating advanced MRI sequences, particularly DWI, into the routine evaluation of intracranial masses in patients with a history of hematologic malignancies.

### Pathological features

Pathological examination of the tumor remains the gold standard for the definitive diagnosis of MS and combines morphology, immunohistochemistry (IHC), and molecular genetic studies. The fresh cut surface of the tumor often exhibits a characteristic greenish or yellowish-green hue due to the presence of myeloperoxidase (MPO), which is the origin of the historical term “chloroma.”. However, this green color is not universal in MS; approximately 30% of cases may exhibit a grayish-white appearance (25). In our case, the fresh cut surface of the tumor specimen appeared grayish-white, which correlates with the weak MPO expression observed on IHC. This correlation is clinically relevant, as it reinforces the principle that gross appearance is an unreliable indicator for MS, and that complete histopathological and immunohistochemical evaluation is mandatory for diagnosis.

Histologically, MS commonly presents with myeloid blasts (myeloblasts). On hematoxylin and eosin (H&E) staining, these cells are pale-stained, medium to large in size, with enlarged and irregular nuclei (often folded), and scanty pale cytoplasm. The nuclei have finely granular chromatin and contain a single small nucleolus, with scanty cytoplasm. The presence of eosinophilic myelocytes, characterized by indented nuclei and moderately eosinophilic orange-red cytoplasm, is considered a highly valuable morphological clue (26). Immunohistochemistry (IHC) is crucial for confirming the diagnosis of MS. According to the 2022 World Health Organization (WHO) Classification of Tumors, Fifth Edition (2), the diagnosis of MS requires support from the following markers: myeloid markers (MPO, Lysozyme, CD68), stem/progenitor cell markers (CD34, CD117, CD133), myeloid differentiation antigens (CD33, CD13, CD15), and other useful markers (CD43, CD99, TdT). The IHC profile in our case (MPO±, CD117+, CD34+, CD68+) fulfills the diagnostic criteria for MS.

Molecular genetic testing plays an increasingly vital role in the modern diagnosis, prognostic stratification, and management of MS. Conventional karyotyping can identify characteristic chromosomal abnormalities; fluorescence *in situ* hybridization (FISH) detects specific gene rearrangements; and DNA sequencing reveals mutations in genes such as FLT3 (including the ITD mutation), NPM1, and CEBPA (27).

### Differential diagnosis

The differential diagnosis for MS requires differentiation from lymphoma, primitive neuroectodermal tumor (PNET)/Ewing sarcoma, rhabdomyosarcoma, and meningioma, among others. Lymphoma cells express specific corresponding lymphoid markers (such as CD20, CD3, CD30) and are negative for MPO (28). Metastatic tumors usually have a definite history of a primary tumor and express corresponding epithelial or neuroendocrine markers on IHC (29). PNET/Ewing sarcoma expresses CD99 and FLI-1, along with specific gene rearrangements such as EWSR1-FLI1 fusion and is MPO-negative (30). Rhabdomyosarcoma demonstrates positivity for myogenic markers (such as Desmin, MyoD1, Myogenin) and is negative for MPO (31). As illustrated in our case, meningioma is radiologically challenging to distinguish from MS, and definitive diagnosis relies on pathological examination. Meningiomas typically express EMA, PR, and Vimentin, but are negative for myeloid-specific markers (32).

### Therapeutic principles

Myeloid sarcoma should be considered an extramedullary manifestation of AML and requires systemic therapy. Treatment should be guided by AML treatment standards, with multidisciplinary comprehensive therapy as the optimal approach. For newly diagnosed MS patients, standard systemic induction chemotherapy regimens should be administered. The efficacy of targeted therapy for MS has not been fully validated. For patients with MS harboring FLT3 mutations, the addition of gilteritinib may provide certain benefits. Furthermore, for AML that relapses in the form of MS, gilteritinib could be a cost-effective alternative to salvage chemotherapy (33). Gemtuzumab has demonstrated efficacy in patients with CD33-positive MS (34). Surgery is not a curative treatment modality but plays a significant role in symptom relief and establishing a diagnosis. The primary objectives of surgery are to obtain tissue for pathological diagnosis, alleviate compression on vital organs, and resect residual lesions following chemotherapy. For MS patients who achieve remission, particularly those with high-risk features, allogeneic hematopoietic stem cell transplantation (allo-HSCT) is a crucial consolidation option. Multiple studies have confirmed that allo-HSCT can significantly improve the long-term survival outcomes of MS patients (35).

Based on these principles, our patient was promptly referred for systemic chemotherapy following gross total resection. The surgical intervention in our case achieved two critical goals: it provided definitive tissue diagnosis and effectively relieved the mass effect, with the patient recovering well postoperatively. However, as surgery alone is not curative for MS, the patient’s subsequent management must be directed by AML treatment protocols. Given the isolated nature of the CNS relapse, the presence of the FLT3-ITD mutation, and the patient’s performance status, we advocate for considering FLT3-targeted therapy (e.g., gilteritinib) as a bridge to allogeneic hematopoietic stem cell transplantation (allo-HSCT). Allo-HSCT remains the most effective consolidation strategy for high-risk extramedullary relapse, particularly in FLT3-mutated AML, and should be strongly considered once the patient achieves a second remission. This case reinforces the principle that management of MS should be delivered in a multidisciplinary setting involving neurosurgery, hematology, radiology, and pathology to optimize patient outcomes.

### Clinical implications and future directions

This case carries several important clinical implications. First, it demonstrates that AML patients remain at risk for extramedullary relapse even after prolonged remission. This suggests that routine surveillance imaging and neurological monitoring may be warranted beyond the conventional follow-up period, although the optimal surveillance strategy remains to be defined. Second, the occurrence of relapse despite standard CNS prophylaxis highlights the need for more effective strategies to prevent sanctuary-site disease, perhaps through novel targeted agents with better CNS penetration. Third, the acquisition of CD56 expression at relapse, which was absent at initial diagnosis, suggests that phenotypic evolution may drive extramedullary migration, raising the possibility that serial monitoring of adhesion molecule expression could identify patients at higher risk. Finally, the role of FLT3 inhibitors in preventing and treating CNS MS warrants further investigation, as these agents may offer a rational approach to targeting the molecular drivers of extramedullary disease.

## Conclusion

We report a rare case of intracranial myeloid sarcoma that occurred as an extramedullary relapse after long-term remission of AML and highly mimicked a meningioma on imaging. This case systematically illustrates the complexities of MS in terms of its epidemiology, pathogenesis, clinical manifestations, imaging features, pathological diagnosis, and therapeutic strategies. For patients with a previous history of hematologic malignancies, clinicians must maintain a high level of suspicion for extramedullary relapse when a new space-occupying lesion appears, even if the radiological findings suggest another diagnosis. Functional imaging modalities (e.g., DWI, PET/CT) provide valuable information for differential diagnosis; nevertheless, histopathological examination remains the diagnostic gold standard. Therapeutically, a multidisciplinary approach centered on systemic chemotherapy (including targeted agents), combined with surgery and allogeneic hematopoietic stem cell transplantation (allo-HSCT), is crucial to achieve optimal outcomes and long-term survival. With the deepening understanding of the pathogenesis of MS and the development of novel therapies, the prognosis for patients with MS is expected to improve further.

## Data Availability

The original contributions presented in the study are included in the article/**Supplementary Material**. Further inquiries can be directed to the corresponding author.
