# Supplementary material for: Intracranial extramedullary relapse of acute myeloid leukemia presenting as myeloid sarcoma mimicking meningioma: a case report and literature review
Source: Front Oncol. 2026 Jul 10;16:1769002. doi: 10.3389/fonc.2026.1769002 (PMC13395718; doi:10.3389/fonc.2026.1769002)
Supplement: Supplementary file 1 [file Presentation1.pptx]

## Slide 1
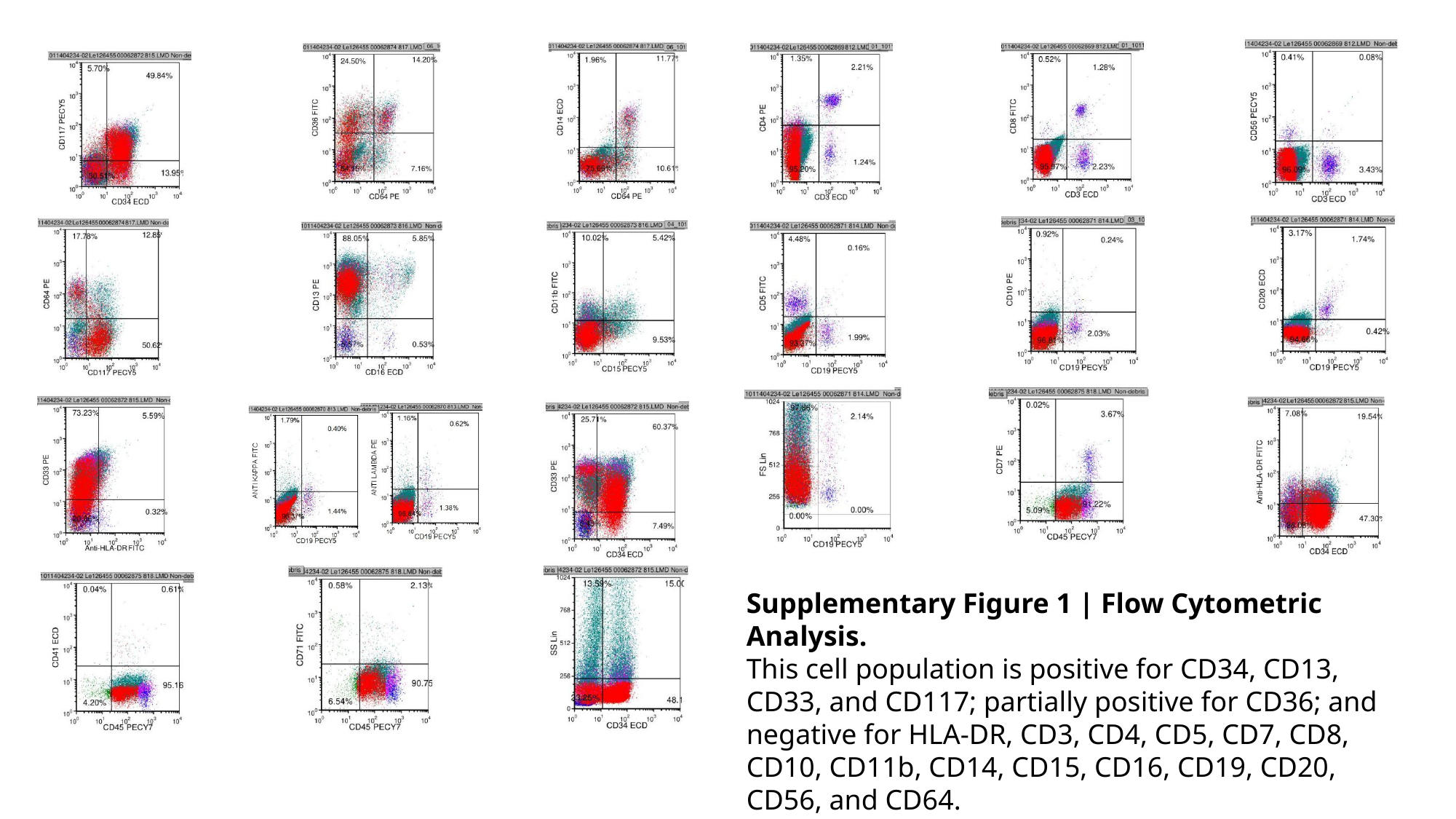

Supplementary Figure 1 | Flow Cytometric Analysis.
This cell population is positive for CD34, CD13, CD33, and CD117; partially positive for CD36; and negative for HLA-DR, CD3, CD4, CD5, CD7, CD8, CD10, CD11b, CD14, CD15, CD16, CD19, CD20, CD56, and CD64.

## Slide 2
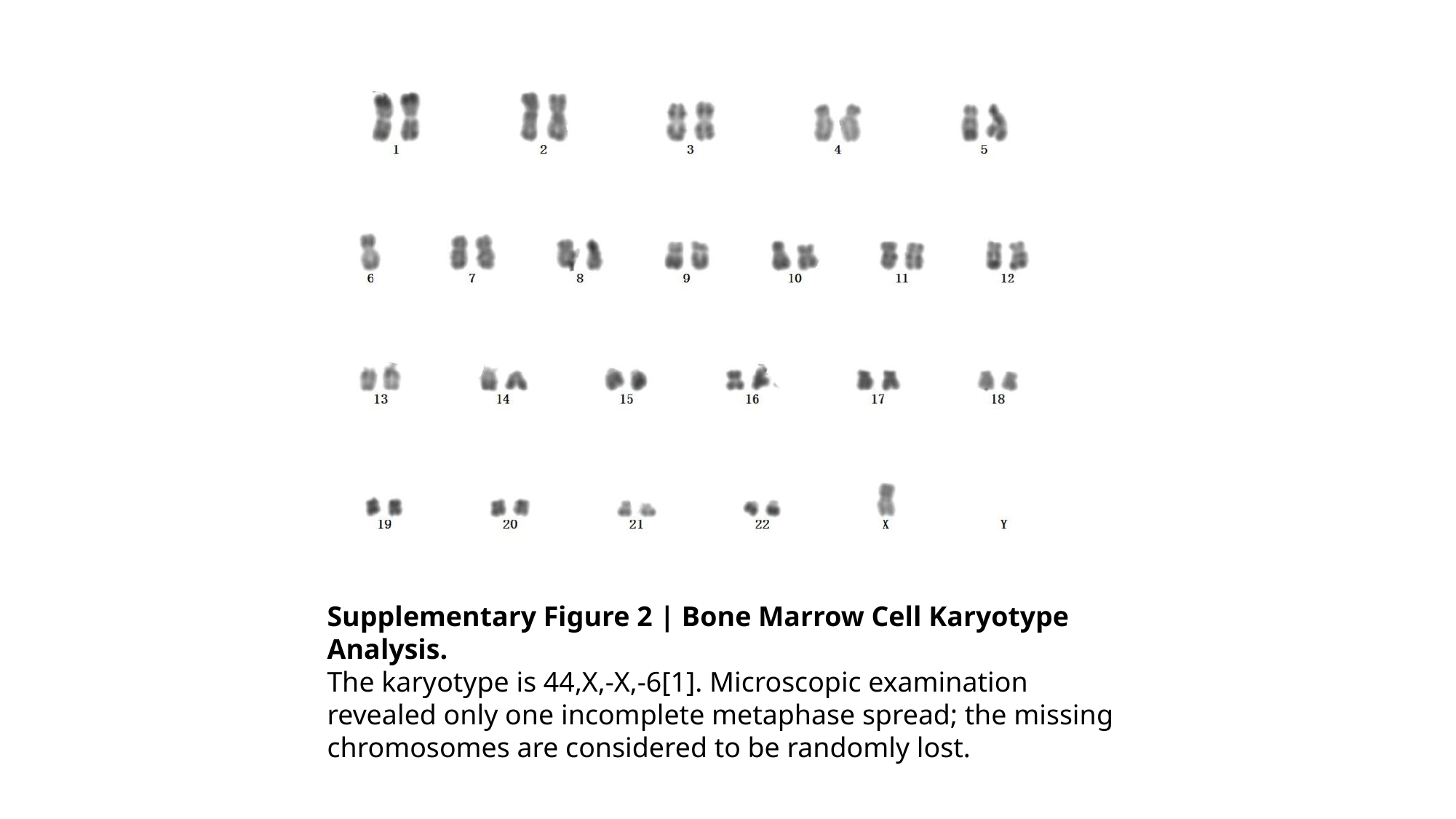

Supplementary Figure 2 | Bone Marrow Cell Karyotype Analysis.
The karyotype is 44,X,-X,-6[1]. Microscopic examination revealed only one incomplete metaphase spread; the missing chromosomes are considered to be randomly lost.

## Slide 3
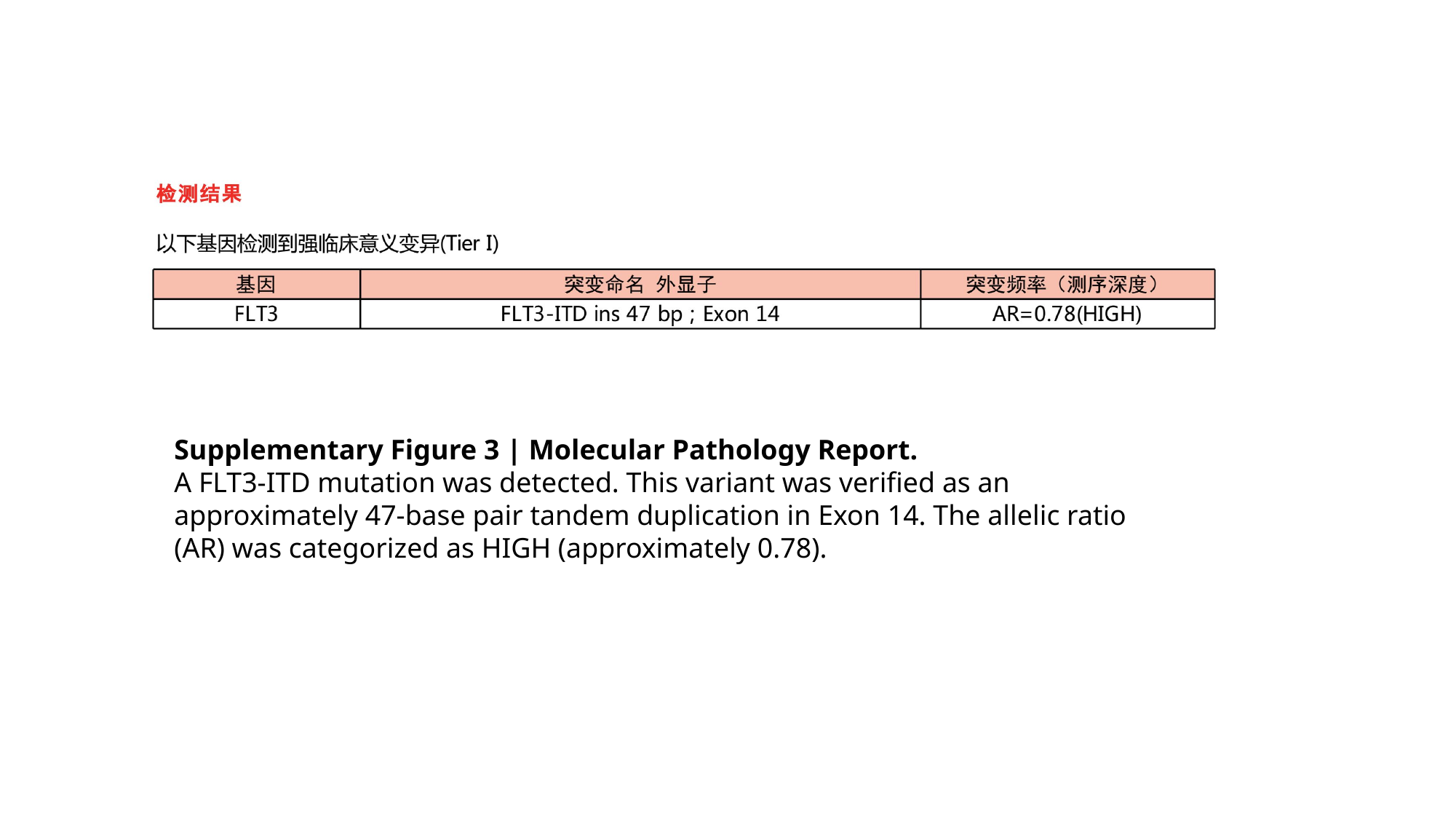

Supplementary Figure 3 | Molecular Pathology Report.
A FLT3-ITD mutation was detected. This variant was verified as an approximately 47-base pair tandem duplication in Exon 14. The allelic ratio (AR) was categorized as HIGH (approximately 0.78).
